# Cycling provision separated from motor traffic: a systematic review exploring whether stated preferences vary by gender and age

**DOI:** 10.1080/01441647.2016.1200156

**Published:** 2016-07-14

**Authors:** Rachel Aldred, Bridget Elliott, James Woodcock, Anna Goodman

**Affiliations:** ^a^ Faculty of Architecture and the Built Environment, Department of Planning and Transport, London, UK; ^b^ Policy Studies Institute, University of Westminster, London, UK; ^c^ UKCRC Centre for Diet and Activity Research (CEDAR), MRC Epidemiology Unit, School of Clinical Medicine, University of Cambridge, Cambridge, UK; ^d^ LSHTM, London, UK

**Keywords:** Cycling, gender, age, equity, systematic review

## Abstract

In this paper, we represent a systematic review of stated preference studies examining the extent to which cycle infrastructure preferences vary by gender and by age. A search of online, English-language academic and policy literature was followed by a three-stage screening process to identify relevant studies. We found 54 studies that investigated whether preferences for cycle infrastructure varied by gender and/or by age. Forty-four of these studies considered the extent of separation from motor traffic. The remainder of the studies covered diverse topics, including preferred winter maintenance methods and attitudes to cycle track lighting. We found that women reported stronger preferences than men for greater separation from motor traffic. There was weaker evidence of stronger preferences among older people. Differences in preferences were quantitative rather than qualitative; that is, preferences for separated infrastructure were stronger in some groups than in others, but no group preferred integration with motor traffic. Thus, in low-cycling countries seeking to increase cycling, this evidence suggests focusing on the stronger preferences of under-represented groups as a necessary element of universal design for cycling.

## Introduction

Within countries with a low cycling mode share (approximately 5% mode share or less, herein referred to as low-cycling countries), cycling is demographically unequal, notably by gender and age (Pucher & Buehler, 2008). A policy concern to diversify cycling has been accompanied by a growth in academic literature on this issue. Aldred, woodcock and Goodman (2015) explored whether increasing cycle commuting (between 2001 and 2011) was associated with greater age and gender diversity in England and Wales. The results suggest that increased cycling in Inner London and some other, largely metropolitan, areas has not yet been associated with an increase in diversity.

Part of the reason for this lack of diversification may lie in a lack of change in existing cycling environments. Increasingly, authors examine the extent to which experience of active travel environments may vary between groups (Asadi-Shekari, Moeinaddini, & Zaly Shah, 2013; Habib, Mann, Mahmoud, & Weiss, 2014; Oxley, Corben, Charlton, Fildes, & Rothengatter, 2005). For example, an ageing population generates new design challenges for cycle infrastructure (Fietsberaad, 2007), while the engineering requirements of three-wheeled cycles (used to carry children or other cargo, or ridden by some disabled cyclists) differ from that of *bi*cycles (Transport for London [TfL], 2014).

Understanding under-represented groups’ views on infrastructure may help realise policy goals to diversify cycling. Specifically, authors have suggested that people from demographic groups under-represented in lower cycling contexts show greater aversion to sharing with motor traffic than do younger people and men (Chataway, Kaplan, Nielsen, & Prato, 2014; Davies, Halliday, Mayes, & Pocock, 1997). If so, this could be part of the explanation for observed inequalities in cycling, especially higher cycling countries, with better cycling infrastructure, have much greater gender and age equity (Aldred et al., 2015).

To date, however, no systematic review has examined gender and age similarities and differences in preferences for different types of cycling environments. This review helps to fill that gap by systematically synthesising the evidence on what people say they would prefer if given a choice. It does not consider the evidence on what people actually choose in existing cycling environments, in which they may have few options. Its findings have policy implications for building infrastructure for cycling in low-cycling countries. They speak to an ongoing debate between those who suggest that building more infrastructure that *existing* cyclists find acceptable will increase and diversify cycling (Office for National Statistics [ONS], 2014) and those who argue that this approach will reinforce existing inequalities (Horton & Jones, 2015).

## Review focus

The paper complements systematic reviews already published in the field of active transport, which focus on intervention research to promote cycling (Yang, Sahlqvist, McMinn, Griffin, & Ogilvie, 2010) or cycle safety (e.g. Owen, Kendrick, Mulvaney, Coleman, & Royal, 2011). One central conclusion of these reviews is that it is hard to draw firm conclusions because of the limited number both of high-quality interventions and of high-quality studies. While several high quality studies have been published subsequently (e.g. Goodman, Sahlqvist, & Ogilvie, 2014; Heinen, Panter, Mackett, & Ogilvie, 2015), the literature remains relatively small.

This evidence gap partly reflects the fact that much transport evidence does not fit neatly into the “intervention” category. Within the topic of infrastructure and cycling uptake, other relevant study types include ecological studies (correlating area-based characteristics with cycling levels, drawing conclusions about the weight of different factors); route choice studies (exploring where current cyclists ride, and deriving “revealed” preferences from this); and stated preference surveys (asking people what infrastructure would encourage them to cycle). The latter form of evidence is the focus of this review which asks whether and how cycle infrastructure preferences vary by gender and age.

The paper joins a growing number of publications in the transport field (e.g. Jothi Basu, Subramanian, & Cheikhrouhou, 2015; Vieira, Kliemann Neto, & Amaral, 2014; Wang & Notteboom, 2014) using a systematic review approach. Although stated preference studies have been common in transport research for some time (Hensher, 1994), they have rarely been synthesised using systematic reviews. Such synthesis is, however, increasingly common in other disciplines that make use of stated preference data, such as health economics (Whitty, Lancsar, Rixon, Golenko, & Ratcliffe, 2014).

Our choice of a systematic review approach means the paper benefits from the increasing robustness that comes with a more comprehensive search. However, the systematic, in-depth approach meant we had to choose a narrower question than narrative reviews can adopt. We would argue that this paper helps to demonstrate the value of systematically reviewing stated preference evidence in transport. We hope that it will be complemented by future systematic reviews of other topics and other types of evidence, including ecological studies and route choice studies.

In this review, an inclusive definition of “stated preference” is used. Traditionally in transport research, stated preference studies refer to techniques specifically used to estimate utility functions, used within choice modelling to predict change in use of transport infrastructure or services and/or to calculate cost–benefit ratios (Kroes & Sheldon, 1988). However, with the field becoming more interdisciplinary, health and social researchers (e.g. Winters & Teschke, 2010) are also conducting research asking about people’s infrastructural preferences, although without the aim of creating utility models. Here, both types of study are included.

## Methods for selection, appraisal and synthesis

Methods are outlined here: for more details on search terms, sources retrieved and screening procedures, please see Appendix. Two authors (RA and BE), the study appraisers, searched the academic databases (EBSCO, Web of Science, ProQuest, PubMed, TRID, ARRB) plus 11 websites (via Google) (end of March 2015), following a search protocol developed by the team with input from additional advisors at the Centre for Diet and Activity Research. We only included studies that covered preferences related to cycle routes and infrastructure; so not, for example, preferences for taking bicycles on trains. Studies were included that reported analysis of any similarities or differences by age and gender. BE screened abstracts and led initial study selection with RA checking wherever uncertainty was flagged. RA appraised the studies.

In the selected articles, separation from motor traffic was by far the most common infrastructural characteristic discussed (with a clear comparison made by age and/or gender in 44/54 studies). This mostly involved questions about the existence or not of some form of separate provision, but sometimes involved questions about motor traffic flows, where sharing takes place. Hence, in analysis, we focused on this issue. Other issues covered were diverse; for example, two studies covering preferences for winter maintenance of cycle infrastructure, and another covering preferences related to “quality of signage”. It was not possible to synthesise similarities and differences related to these issues.

There are no established reporting guidelines for stated preference studies. We extracted data from each study on (i) issues that affect internal validity, (ii) issues that affect external validity or generalisability, and (iii) sample size. For internal validity, we focused on how preferences were elicited. Where little detail is given, participants may imagine quite different kinds of infrastructure when responding. Specifically, a “cycle lane” may be imagined as being effectively shared with motor traffic (an advisory painted lane), or separated by bollards, kerb or other barriers. More detail may allow more discrimination between different levels of separation from motor traffic.

- How situations were communicated to participants, for example, words only, images, video.
- How specific the situations presented to participants were categorised as follows:
  - Low to very low specificity, for example, respondents choosing between “cycle lane present”, and “no cycle lane”.
  - Medium specificity, for example, respondents asked to choose between on-road segregated infrastructure, painted cycle lanes, and off-road tracks.
  - High specificity, for example, images of a range of different infrastructural types with differing degrees of separation from motorised traffic.

We considered external validity to refer to whether survey results represent broader population views about preferred cycling environments. We did not consider the wider issue of whether these stated preferences accurately predict subsequent behaviour change (Bradley, 1988) because we would argue that views about desired service provision are important in themselves.

Sampling methods were categorised as follows:

- Convenience sample, for example, students, participants in cycle touring event.
- Purposeful convenience sampling, for example, potential cyclists, employees.
- Representative survey, for example, randomly sampled national travel survey.

Studies with higher quality sampling methods of the general population are more likely to be representative of a potential cycling population. Inclusion of non-cyclists^1^ was considered important as there are suggestions in the literature that cyclists’ preferences, particularly in low-cycling contexts, may not represent the views of potential cyclists (Horton & Jones, 2015).

Three rounds of screening were carried out to filter the evidence, with data extracted into a bespoke table in Excel. Analysis in Excel and SPSS explored both headline findings (similarities and differences in preferences) and the extent to which these were associated with study design. We attempted to record information that could be used for meta-analysis – quantitatively combining the results from multiple studies – but in general, information such as sub-group means was not provided, meaning that meta-analysis was not possible.

## Results

### Studies included and excluded

**Figure 1.**
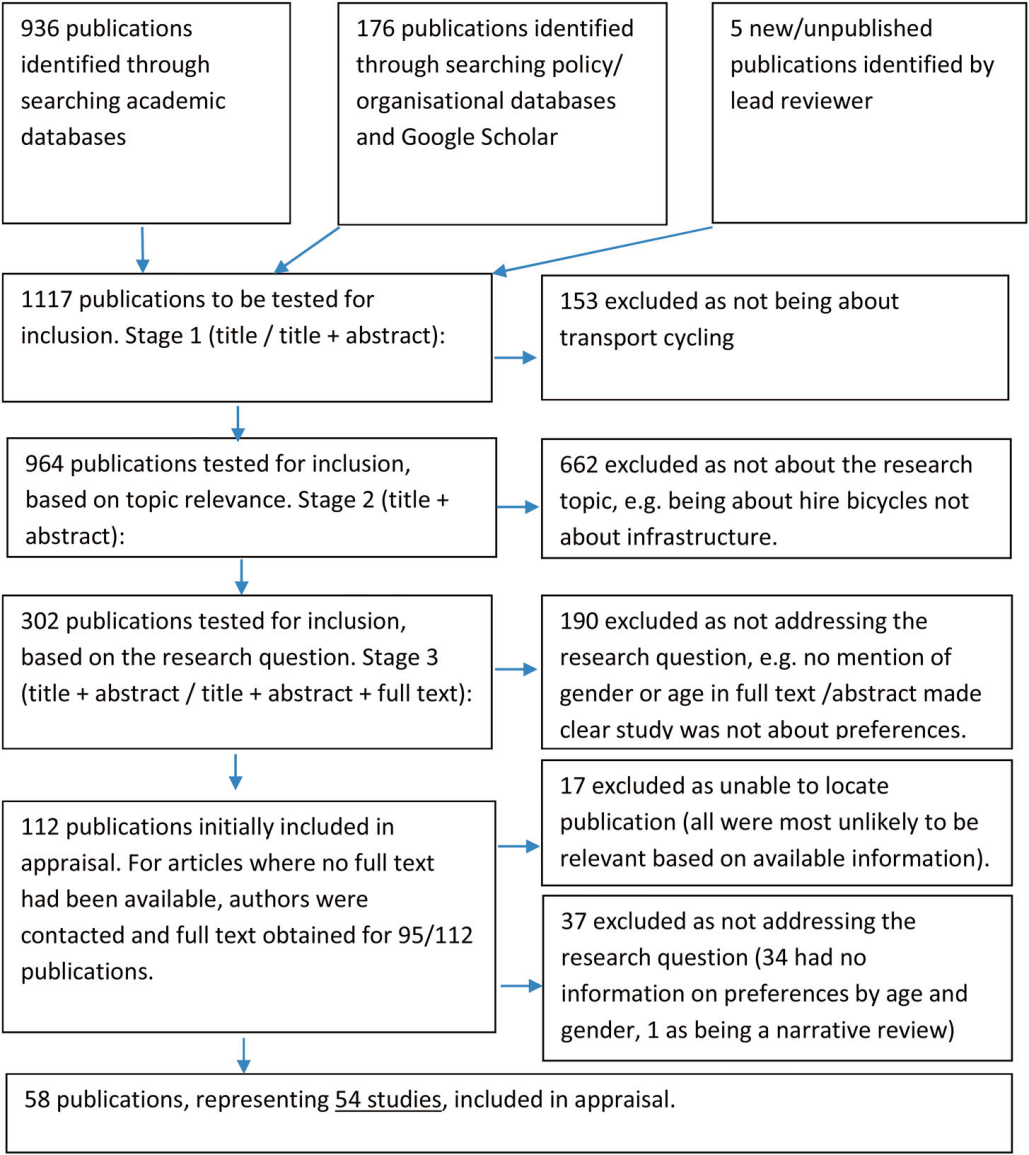
Summary of evidence management strategy.

Our search strategy led to the identification of 54 separate studies, reported in 58 publications (Figure 1).^2^

#### About the studies

**Figure 2.**
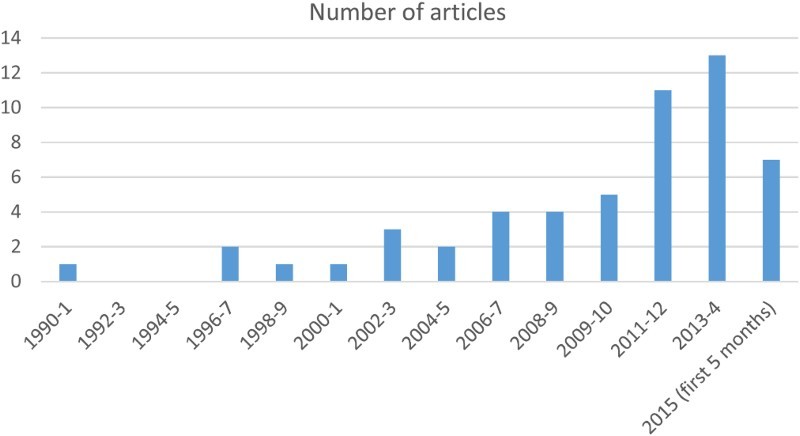
Articles by year.

Fifty studies examined stated preferences in relation to gender, with 33 covering age (adults) and only 2 discussing preferences related to child cycling (Table 1). A summary of study characteristics is presented in Table 3 and Figure 2.

**Table 1.** Characteristics of included studies.

		Number of studies	Percentage of studies
Sample size	Under 50	2	4
	50–99	6	11
	100+	45	83
	Not stated	1	2
Study composition	All cyclists, or under 50% non-cyclists	39	72
	At least 50% non-cyclists	12	22
	Not stated	3	6
Country of origin	USA	19	35
	UK	8	15
	Belgium	3	6
	Canada	4	7
	Other/more than one country	20	37
Sampling method	Convenience sample	34	63
	Purposive convenience sampling	5	9
	Random sampling	14	26
	Not stated	1	2
Preference elicitation method	Description (only)	26	48
	Existing infrastructure	8	15
	Images	16	30
	Video	4	7
Situational specificity	Very low	25	46
	Medium	19	35
	High	10	19

It can be seen that this is a growing field, with 2009–2010 onwards providing a steady increase in the numbers of studies published. There is the potential to benefit from this growth by developing more consistent measures and/or sharing data for meta-analysis.

As noted above, the synthesis below includes 44 of 54 studies, with patterns similar to those for all 54 studies in terms of study composition and so on.

#### Country of origin

Over one-third of all studies were conducted in the U.S.A (19 studies), with eight from the UK, followed by Belgium and Canada (four each). Over two-thirds (39 of 56) were carried out only in high-income countries with low cycling rates. In terms of classifications, Australia, Brazil, Canada, Ireland, New Zealand, Spain, UK and U.S.A were judged to be low cycling. Other countries were judged to be medium or high cycling (Belgium, China, Denmark, India, the Netherlands and Sweden).

#### Study size and populations

Sample size varied considerably (35–3494, with one not stated). Most studies included more men than women. This was particularly true in studies set in low-cycling countries and drawing their sample from existing cyclists (in one study, “avid cyclists”). Only in two-fifths of the studies, at least 20% of the sample were non- or infrequent cyclists. A little over one-third of studies only sampled cyclists, while one only exclusively sampled non-cyclists. Overall, the proportion of regular cyclists included was far higher than for the general population, this being particularly true in studies in low cycling countries using convenience samples.

#### Reporting of results

Results were reported in diverse ways; for example, scores given out of five to different infrastructure types, or percentage of people agreeing that they would use a particular type of cycle route. Given the information available, a meta-analysis was not possible. For example, 13 of the 17 studies that reported no statistically significant gender differences in preferences for separation did not give subgroup means.^3^

#### Sampling and elicitation methods

Sampling methods varied widely from household surveys to convenience samples of cyclists attending specific rides. Nearly two-thirds used convenience sampling with around a quarter of studies using random sampling.

Various study methods were used to elicit preferences (see Table 1). Almost half gave a text-based description of an infrastructure type (e.g. “painted lane”), conducted either using a paper questionnaire, on the phone, in person, or online. The participant would then be asked to rate the infrastructure type, although the type of rating would depend on the survey: including ranking preferences, assigning hypothetical monetary values, or asking people whether they would feel comfortable or safe.

The second most common type of elicitation method was to use images, either real or computer-generated. These were accompanied by questions about the desirability of the infrastructure type, as with studies using text-based elicitation. A less common method referenced existing infrastructure; for example, one study stopped cyclists in a series of sampled cycle lanes and asked them to rate the lane compared to other types of infrastructure. In other cases, researchers showed participants videos of infrastructure types, and then asked about preferences.

Finally, the situational specificity of the survey questions varied (see Table 1). Nearly half were very general (e.g. asking about “cycle lanes”) with one in five very specific, for example, testing a range of infrastructure types with differing extents of segregation. The remainder were in between, for example, making trade-offs between different infrastructure situations and trip times; with situations including bus/cycle lanes, parks/quiet residential streets (combined option), on road cycle lane, and off-road track.

### Infrastructural preferences

#### Findings: gender and preferences for greater segregation from motor vehicles

**Figure 3.**
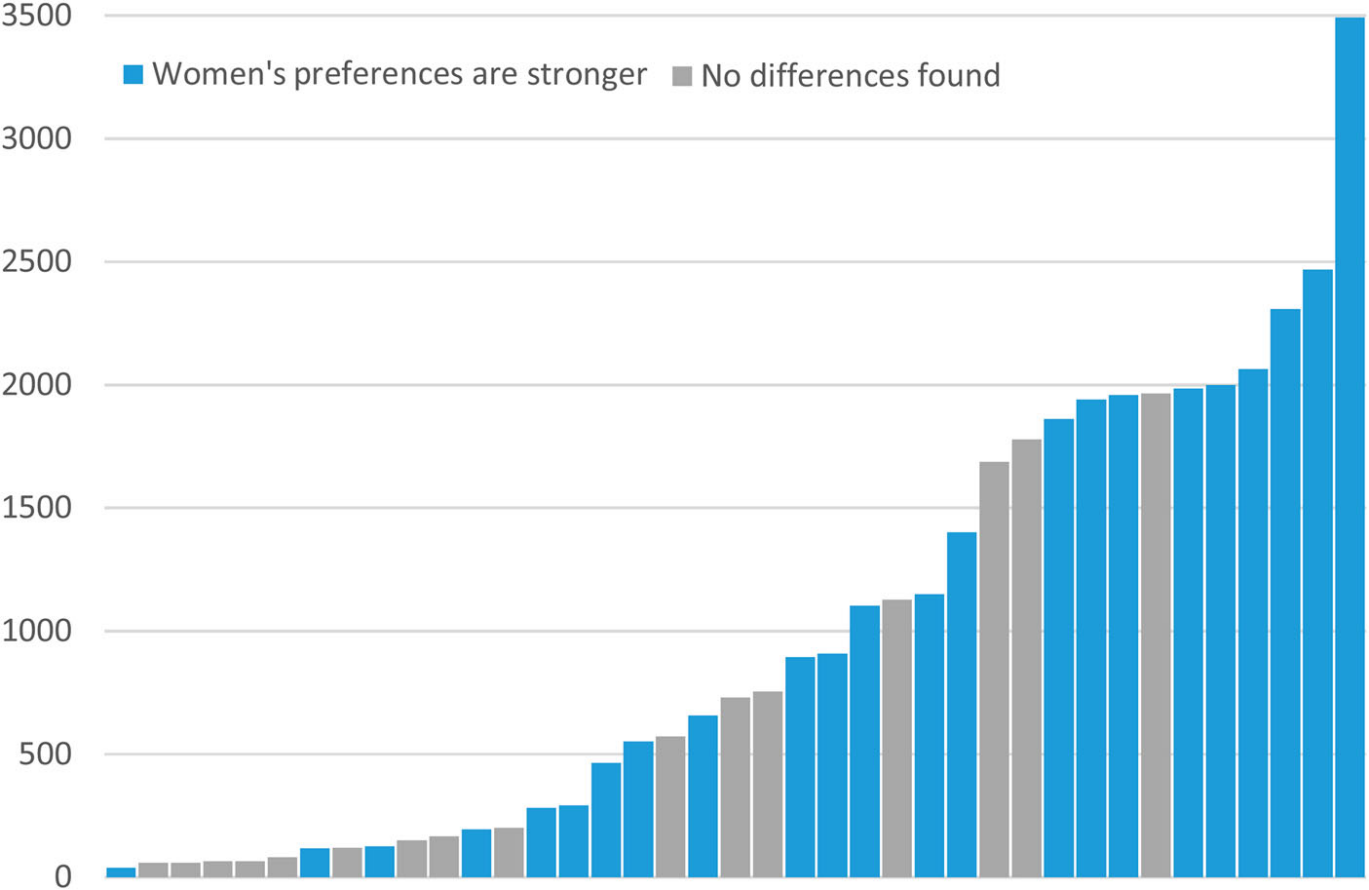
Gender and preferences for separated infrastructure, by sample size (minus one study with missing sample size).

Forty studies provided evidence as to whether preferences for separation from motor traffic differed by gender. Of these, 23 (57.5%) said women expressed stronger preferences for segregation from motor vehicles than did men (Table 2). Seventeen studies (42.5%) reported no statistically significant differences in gender preferences. No studies reported that men had stronger preferences than women for greater segregation from motor vehicles. Most studies that found no gender difference were small, and likely to have been insufficiently powered (see Figure 3) to detect a relevant difference. Among studies containing at least 200 participants, 20/29 (69%) reported stronger preferences in women than in men, whilst amongst studies containing fewer than 200 participants, only 3/10 (30%) did so.^4^

**Table 2.** Preferences for separated infrastructure by age and gender.

	Preferences for separated infrastructure by gender and age	Number of studies	Percentage of studies
Gender	Women’s preferences are stronger	23	57.5
	No statistically significant differences	17	42.5
	Men’s preferences are stronger	0	0
Age	Older people’s preferences are stronger	9	38
	No statistically significant differences	12	50
	Younger people’s preferences are stronger	3	13

Four-fifths of studies that found differences in preferences between men and women (19/24) also highlighted overall similarity in preferences across genders. For example, for both sexes, more people preferred complete separation from motor traffic compared with the presence of a painted lane but the gap in women was larger.

Differences were found by study type and composition. Smaller studies were less likely to report a gender difference, and some may have been underpowered to detect a meaningful difference. Of studies with larger sample sizes (> 100) and at least 20% non- or infrequent cyclists, 76.5% (17) found gender differences against 23.5% (4) who did not.

Of studies providing a high level of specificity, 78% (*n* =7/9) found a gender difference; this proportion was lower for studies with medium or low specificity (54% and 50%, respectively), but the difference was not statistically significant (*p* = .37 for trend). Studies that contained at least 50% non-cyclists found gender differences in 58% (7/12) of cases, while studies that did not found gender differences in 62% (16/26) of cases (*p* = .85 for difference).^5^

By contrast, study context made a difference to findings. Among studies conducted in low-cycling countries, 69% (*n* = 20/29) found gender differences in preferences for separated infrastructure, while only 27% (3/11) found differences in studies where some or all participants lived in medium- or high-cycling countries (chi-squared *p* = .02 for association).

#### Findings: age and greater segregation from motor vehicles

**Figure 4.**
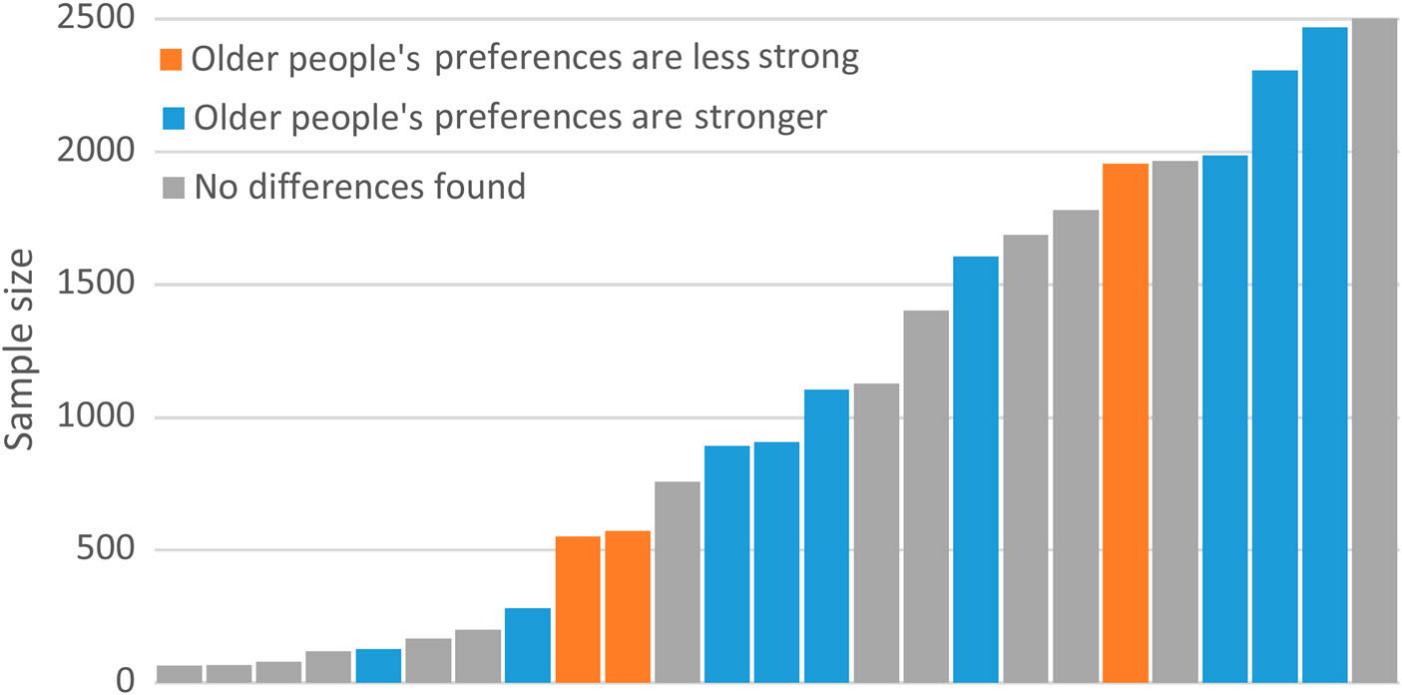
Age and preferences for separated infrastructure, by sample size.

Only 25 studies reported on age, with findings less consistent than for gender. While 9 studies (36% of those reporting on preferences for greater segregation and age) found that older people expressed stronger preferences for separation from motor vehicles, 13 (52%) found no differences, and 3 (12%) reported that older people had less strong preferences for separation from motor vehicles than younger people (Figure 4). Twenty-two out of 25 studies covering older people’s preferences highlighted overall similarity in preferences across age groups.

The relationship between sample size and findings is less clear-cut than for gender, although smaller studies were more likely to find “no difference” (4 out of 5 of studies with a sample size of below 200, compared to 9 out of 20 for the larger studies).

It might be thought that (among participants who cycle) older cyclists’ preferences do not stem from age per se, but from their having likely cycled longer than younger participants. We did not find support for this: among studies that mentioned controlling for cycling experience, an independent “age effect” remained in at least some of these, although reporting was sometimes unclear. However, some studies examining experience found an independent “experience effect” shaping perceptions of cycling infrastructure instead of, or as well as, age and gender effects (e.g. Ma & Dill, 2015; Ma, Dill, & Mohr, 2014).

#### Findings on child preferences or adult preferences for riding with or by children

Because we only found two studies addressing preferences for infrastructure involving children (Aldred, 2015 and Ghekiere et al., 2015), no attempt was made to quantitatively synthesise these. The former compared adults’ preferences for infrastructure for themselves when riding alone, to their preferences for themselves when riding with children, or when deciding whether a child should cycle. The latter compared adults’ preferences for child cycling with the children’s own preferences. Both studies point to a stronger preference for separation from motor traffic where children are cycling. This goes beyond barrier separation and covers issues such as, in Aldred (2015), protection at crossings and reduction in rat-running (when drivers use residential streets as a short cut avoiding main roads), and in Ghekiere et al. (2015), the need for wide and even cycle paths.

The Table 3 summarises some key points from the literature on preferences for infrastructure separated from motor traffic, as synthesised in the tables above:

**Table 3.** Gender, age and infrastructure preferences.

Citation key	Country	Population	Statistically significant differences reported	Similarities
Akar, Fischer and Namgung (2013a)/Akar, Fischer and Namgung (2013b)	USA	Ohio State University students, faculty, staff	Females are more likely than males to self-identify as a “beginner cyclist who prefers to stick to the bike trails, paths and/or sidewalks” while substantially more males self-identified as an “advanced, confident cyclist who is comfortable riding in most traffic situations”. Proportions of female students who chose “vehicular traffic” and “lack of bicycle lanes/paths/trails” as barriers to bicycling were significantly higher than that of the male students	
Aldred (2015)	UK	Mostly cyclists in UK	Study asked whether 10 aspects of the cycling environment would be suitable for riding on by (a) “most people” (b) on their own (c) carrying a child (d) with an 8-year-old (e) by a 12-year-old. Seven of these 50 differences were significant, with more support for segregation among females	For 43 out of 50 situations, there were no significant differences. In particular, there were no significant gender differences for questions related to 8- and 12-year-olds
Antonakos (1995)	USA	552 cyclists at four recreational bike tours in Michigan	For commuter cycling preferences, females rated bike lanes and bike paths higher on average, than males. On a scale from 1 (not at all preferred/not at all important) to 5 (very preferred/extremely important), females rated bike lanes and bike paths at 4.2 and 4.1, respectively, on average compared to 3.9 and 3.6 for males. Females and males both rated traffic safety as high importance, though females gave higher ratings (4.5 vs. 4.1)	The hierarchy of “corridor type” preferences was similar with regards to commuting: males and females both rated bike lanes and wide curb lanes as high preferences
Antonakos (1995)	USA	552 cyclists at four recreational bike tours in Michigan	For recreational cycling preferences, cycling experience and age were negatively associated with preference for bike paths, sidewalks, dirt roads, and trails for recreational cycling For commuting cycling preferences, age and cycling experience were negatively associated with preference for bike paths, sidewalks, and dirt trails for commuting	Age was not associated with concerns about traffic and safety
Berggren, Graves, Pickus, and Hand Wirtis (2012)	USA	Portland cyclists	Females more often responded “much more likely” over “somewhat more likely” for every street characteristic by at least 9%. Safety characteristics of a bike route (e.g. markings, low traffic, traffic lights at arterials, etc.) were more important to female cyclists	Survey data did not indicate large gaps between the preferences of male and female cyclists when combining those who were “much more likely” and “somewhat more likely” to select a route based on a characteristic
Bernhoft and Carstensen (2008)	Denmark	Pedestrians and cyclists aged 40–49 and 70+ in two provincial cities in Denmark	A significantly higher proportion of females than males found cycle paths important for their comfort In the younger group, a higher proportion of females than males would choose a route with cycle path and signalised crossings	Presence of cycle paths was the most important route attribute for both men and women
Bernhoft and Carstensen (2008)	Denmark	Pedestrians and cyclists aged 40–49 and 70+ in 2 provincial cities in Denmark	A higher proportion of older than younger respondents said that the presence of cycle paths was most important for their comfort The amount of traffic was not as important for the younger group as for the older group	Both the older and younger cyclists felt that the presence of cycle paths was most important for their comfort (over 80% for both groups)
Börjesson and Eliasson (2012)	Sweden	Cyclists in Stockholm		Males and females placed equal value on the different route types, for example, cycling time spent on street (or on a cycle path)
Börjesson and Eliasson (2012)	Sweden	Cyclists in Stockholm		The marginal utilities of time and money were not significantly dependent on age
Brick, McCarthy, and Caulfield (2012)	Ireland	Cyclists and non-cyclists in Dublin	Females had a greater preference than males for “greenways” and “off road cycle lanes”	Ordering of route types was the same for both genders – off-road cycle lane, then Greenway, then on-road cycle lane, then shared bus lane, then no facilities
Deenihan and Caulfield (2015)	Ireland	Tourists at two locations in Dublin	Male tourists were more likely than females to choose a road without cycling facilities, with female tourists more likely to choose a road with cycle lanes or a segregated from traffic cycling facility	Female tourists were very unlikely to select a road without any cycling facilities
Deenihan and Caulfield (2015)	Ireland	Tourists at two locations in Dublin	Younger tourists were more likely than older tourists to choose a road without any cycle infrastructure. Older tourists have a higher preference for a fully segregated facility, over a cycle lane	
Chataway et al. (2014)	Australia/Denmark	Cyclists targeted through university networks and cycling forums in Brisbane and Copenhagen. Fliers left on bikes in Copenhagen	Male cyclists were linked to lower fear of traffic (measured through a range of questions about cycling situations) in comparison with female cyclists	
Chataway et al. (2014)	Australia/Denmark	As above	Older cyclists were more averse to riding in mixed traffic	
dell’Olio, Ibeas, Bordagaray, and Ortúzar (2014)	Spain	117 self-classified potential bike users in Santander	Women were more critical than men of existing cycle networks, placing more value on adequate and safe paths	The influence of “existence of an adequate cycle path” in the mode choice model was not affected by gender
Dickinson, Kingham, Copsey, and Pearlman Hougie (2003)	UK	Employees at three organisations in Hertfordshire	Cycle paths were particularly popular among females who lived near enough to cycle and had access to a cycle	Cycle paths were popular amongst both males and females
Dill and McNeil (2014)	USA	Residents in Portland	Females were under-represented among more confident adults and those who cycled for transportation	
Dill and McNeil (2014)	USA	Residents in Portland	Older adults were under-represented among more confident adults and those who currently cycle for transportation	
Dill, Goddard, Monsere, and McNeil (2015)	USA	Cyclists and residents in five large US cities (Cyclists and non cyclists)	On several measures of safety and comfort, female cyclists using protected bike lanes had more positive associations with the lanes than males. For example: - Females were more likely to agree that the new protected bikeways were safer than other city bikeways (93% vs. 87%) - Females were slightly more comfortable than males on paths or trails separated from the street and less comfortable on commercial streets without bike lanes For potential female bicyclists (part of the residential group), protected lanes increased stated comfort levels significantly, though females still reported lower comfort levels than males Among residents interested in bicycling more for transportation, 87% of females and 82% of males agreed that they would be more likely to ride if motor vehicles and bicycles were physically separated	Both males and female intercepted cyclists overwhelmingly felt that protected bike lanes increased their safety while riding in them There were no gender differences found amongst intercepted cyclists in relation to some statements about protected bike lanes, for example: - The buffer section with parked cars makes me feel safe (asked on lanes with buffers) - The buffer effectively separates bikes from cars - The buffer does a good job of protecting bikes from cars - The lane design effectively separates bicyclists from pedestrians Amongst intercepted cyclists, there were few gender differences with regards to facility design
Emond, Tang, and Handy (2009)	USA	Random sample of residents in six small cities in the USA	Males were more likely than females to report that they would ride with heavier traffic, despite similar levels of discomfort	Males experienced approximately as much discomfort on average as females on facilities not separated from heavier traffic
Gardner (1998)	UK	Leisure cyclists, non-cyclists and utility cyclists in different areas of England	More males than females were willing to cycle alone, with some females indicating that they did not feel safe if cycle lanes were too isolated, or in parks or the countryside on their own	
Heesch, Sahlqvist, and Garrard (2012)	Australia	Adult cyclists in Queensland who were members of Bicycle Queensland (BQ) club	For transport cycling, females were less likely than males to prefer cycling on the road For recreational cycling, females were less likely than males to prefer cycling on-road, but more likely to prefer cycling off-road Top constraints for at least half of males and females were perceived environmental factors, namely traffic and aggression from motorists, with females significantly more likely to report these	For transport cycling, few males or females preferred to cycle on the road On-road routes were even less preferred for transport cycling than recreational cycling by both males and females
Hughes and Harkey (1997)	USA	Twenty-three casual and 12 experienced cyclists	Apparent reduction in sensitivity to risk on the part of the “younger cyclist” (under 20 years)	
Hunt and Abraham (2007)	Canada	Cyclists in Edmonton		Indications that older people had less aversion to mixed traffic and that the very young had less aversion to paths were statistically weak
Krizek, Johnson and Tilahun (2005)	USA	292 current and potential cyclists in Minnesota	Females were willing to travel more additional minutes than men for a preferred facility. Assuming a 20 minute commute, males were willing to divert 5.43 fewer minutes than females	Male and female cyclists were relatively similar in the proportion who valued specific facilities such as on-road lanes, separate paths, and a connected system of bicycle routes Both males and females were willing to travel longer for an off-road facility, followed by a bicycle lane with no on-street parking
Landis, Vattikuti, and Brannick (1997)	USA	150 cyclists aged 13+ in Tampa, Florida		No significant difference in mean bicycle quality-of-service scores by gender
Landis et al. (2003)	USA	60 cyclists aged 13+ in Orlando, Florida		No significant difference in mean bicycle quality-of-service scores by gender
Lawson, Pakrashi, Ghosh, and Szeto (2013)	Ireland	1954 cyclists, who regularly cycled in Dublin within the previous 12 months		For both genders, cyclists who preferred to use roads with no cycling facilities were more likely to describe cycling as safe than others
Lawson et al. (2013)	Ireland	As above	The probability of describing cycling as safer than or as safe as driving grew with age	
Li, Wang, Liu, Schneider, and Ragland (2012)	China	805 cyclists in the metropolitan area of Nanjing		Male and female bicyclists did not perceive different levels of comfort on physically separated bicycle path sections
Li et al. (2012)	China	805 cyclists in the metropolitan area of Nanjing	Bicyclists under 30 were 10% more comfortable on average across all facilities studied than those over 30	
Lusk, Wen, and Zhou (2014)	China	1150 adults in Hangzhou	The difference between males and females preferring to use the road was statistically significant (5.3% male, 3.0% female) Females preferred segregated cycle tracks more than males (60.2% vs. 53.9%) Females preferred bicycle signals more than men (69.1% vs. 63.7%)	Few males or females preferred to use the road Preference for bicycling on segregated cycle tracks in the study population was almost double in both genders, compared to all other types of route
Ma and Dill (2015)	USA	Random phone survey of 902 adults in Portland, Oregon region	Females with children were more likely to perceive their relatively “high bikeable” neighbourhoods as low bikeable, than males with children	
Ma and Dill (2015)	USA	Random phone survey of 902 adults in Portland, Oregon region	Compared with people aged 18–34, middle-aged (35–54) people are less likely to hold low perceptions in “high bikeable” neighbourhoods; while older people (55+) are nearly three times more likely to perceive “high bikeable” environments as low	
Majumda, Mitra, and Pareekh (2015)	India	Residents of two small Indian cities	Significant gender differences were found for the following statement for one of the samples: I will not cycle because of safety hazard associated	No significant gender differences were found for the following statement for both samples: - I will not cycle because presence of other motorised vehicles makes it difficult for bicycle commuters especially in peak hours
Majumda et al. (2015)	India	Residents of two small Indian cities		No significant age differences were found for the following factors/statements for both samples: - I will not cycle because presence of other motorised vehicles makes it difficult for bicycle commuters especially in peak hours - I will not cycle because of safety hazard associated
Mertens et al. (2014)	Belgium	66 Flemish adults (45–64 years) living in an urban (>600 inhabitants/km2) or semi-urban (300–600 inhabitants/km2) municipality in Flanders or Brussels Capital Region		No moderating effects of gender or age when exploring environmental factors related to the invitingness for transportation cycling
Misra et al. (2015)	USA	127 users of Cycle Atlanta smartphone application	Gender had an influence on rider type self-categorisation: - Male cyclists were more likely to categorise themselves as “strong and fearless” or “enthused and confident” than the female cyclists; - Females were more likely to classify themselves as “comfortable but cautious” than male riders	Riders across all cyclist types prefer dedicated cycling facilities and are opposed to high-speed traffic and high-volume traffic
Misra et al. (2015)	USA	As above	Age had an influence on rider type self-categorisation: - Older people were less likely to categorise themselves as “strong and fearless” - With increasing age people are more likely to group themselves into less confident groups	As above
Parkin, Wardman, and Page (2007)	UK	144 cyclist and non-cyclists from Bolton Metropolitan Borough Council, the University of Bolton and Bolton Royal Hospital	Males generally considered cycling more acceptable than females	Models were estimated that included person type variables and interactions between those and journey variables. While these did show some significant effects, they were often at the expense of the main effects becoming non-significant
Parkin et al. (2007)	UK	144 cyclist and non-cyclists from Bolton Metropolitan Borough Council, the University of Bolton and Bolton Royal Hospital	The young and older people perceived junctions as adding more risk than those aged 35–44 Young and older people generally considered cycling less acceptable than those aged 35–44	
Petritsch, Ozkul, McLeod, Landis, and McLeod (2009)	USA	80 cyclists at the Ride for Science 2009 in Tampa, Florida		No statistically significant grading difference was found between genders in this bicycle level of service study of paths adjacent to roadways
Petritsch et al. (2009)	USA	80 cyclists at the Ride for Science 2009 in Tampa, Florida		No statistically significant grading difference was found between age groups
Ryley (2005, 2006)	UK	Cyclists and non-cyclists in West Edinburgh	More females than males said that more money should be spent on on-road cycle lanes, and that safety fears prevent them from cycling The model coefficient for “facilities on route” was higher for women than males	
Sallis et al. (2013)	USA	1780 adults aged 20–65 in Seattle, Washington and Baltimore, Maryland regions		No association between gender and the stated projected increase in cycling if safety from cars was improved
Sallis et al. (2013)	USA	As above		No association between age and the stated projected increase in cycling if safety from cars was improved
Sanders (2014)	USA	263 people who drive and/or cycle, in the San Francisco Bay Area	Female respondents were significantly less comfortable than males sharing with motor traffic	Barrier-separated bicycle lanes were popular among potential and current cyclists, irrespective of gender, age, and cycling frequencies
Segadilha et al. (2014)	Brazil	65 (80% male) cycle commuters in a medium-sized Brazilian city (São Carlos, SP)		No statistically significant gender differences in factors influencing cyclist route choice
Segadilha et al. (2014)	Brazil	As above		No statistically significant age differences in factors influencing cyclist route choice
Sener, Eluru, and Bhat (2009)	USA	1605 cyclists across more than 100 cities in Texas	Male bicyclists perceived bicycle facilities in their community to be better than did female bicyclists	
Sener et al. (2009)	USA	1605 cyclists across more than 100 cities in Texas	Young bicyclists (18–24) had the most positive perception of safety from traffic crashes Young bicyclists perceived bicycle facilities in their community to be better than did older bicyclists Bicycle lanes led to a substantial improvement in perception of safety from traffic crashes, particularly for individuals aged 65 +	No statistically significant difference in safety perceptions among individuals of different ages beyond 24 years
Steer Davies Gleave (2010a, 2010b)	UK	Cyclists and non-cyclists in London	Females gave “No traffic on the road”, “Segregated cycle lane” and “Junction types” higher ranks than did males, while “Unsegregated cycle lane” was lower down the list compared to males On average, females rated the absence of (clearly marked) cycle lanes as more unsafe than males. For example, the mean safety score given for “no cycle lanes” varied between 7.3 and 7.8 for different female age groups, compared to 6.5 to 7.1 for different male age groups (where 1 is “completely safe” and 10 “completely unsafe”)	Males and females both had stronger preferences for segregated cycle lanes (compared to no cycle lane or non-segregated cycle lanes) and no traffic (compared to low volume of traffic) Men and women of all ages had similar infrastructural preferences, on average, when comparing kerb-segregated and off-road lanes (the two most preferred infrastructure types), clearly marked cycle lanes (less preferred) and no cycle infrastructure (least preferred)
Steer Davies Gleave (2010a, 2010b)	UK	Cyclists and non-cyclists in London	For females, perceptions of safety generally decreased with age, particularly aged 55–64 and 65 + The youngest males tended to rate the choices as less safe than those in other age groups, although not as much as the oldest group	As above
Steer Davies Gleave (2012)	UK	2307 cyclists in London	There was a significant difference in confidence between males and females saying that they felt confident to cycle on all roads (79% vs. 50%) Females were much more likely to prefer routes away from other traffic and difficult junctions. The average score for “Safety is the most important consideration when choosing a cycle route” for females was 0.89, compared to 0.53 for males (based on a five point scale from strongly agree (+2) to strongly disagree (-2)) In general, female respondents were slightly more likely to rate each junction as less safe than males and more prepared to detour Though male respondents agreed on average that they would avoid a route with difficult junctions, they were less certain they would avoid that particular route (0.66 compared to 1.04 for females) Female cyclists were slightly more willing to change their route in order to use a dedicated on-road cycle lane (56% vs. 48%)	Generally, “route choice considerations” were in the same direction (where >0 indicates agreement and >0 disagreement) There was no variation between male and female cyclists in willingness to consider changing routes to use a cycle superhighway
Steer Davies Gleave, 2012	UK	2307 cyclists in London	Those aged 55 or over or under 35 were more likely to choose to cycle on routes with less traffic (or in a separate cycle lane) Greater willingness to change route for parks and green spaces amongst over 55s: 67% said that they would change their route, compared to 58% of 35–54-year-olds, and 47% of under 35s At junctions, older respondents reported feeling less safe than younger ones and slightly more prepared to detour Willingness to consider changing routes to use a cycle superhighway increases slightly with age	Willingness to change route for a dedicated on-road cycle lane differed little by age group
Stinson and Bhat (2003)	USA	3145 individuals in Texas (mostly avid bicyclists who use computers)	Older respondents associated a higher disutility for routes with car parking; however, as for roadway class, the impact of age was small Older people had a marginally higher preference for wide near-side lanes, and disliked major intersections more than younger people	The differential preference for residential streets (compared to minor arterials) is small: even for a cyclist 100 years old, the magnitude on the minor arterial coefficient drops from –0.77 to –0.69 Variations across individuals are marginal compared to the main effects
Tilahun, Levinson, and Krizek (2007)	USA	167 employees from University of Minnesota, excluding students and faculty		Gender was not significant for probability of choosing a higher quality route
Tilahun et al. (2007)	USA	As above		Age was not significant for probability of choosing a higher quality route
Tin Tin et al. (2010)	New Zealand	2469 cyclists, aged 16+, enrolled in the 2006 Wattyl Lake Taupo Cycle Challenge	Female cyclists were more likely to report the importance of all factors in encouraging their cycling, including more bicycle paths and lanes	Men and women both rated bicycle lanes and bicycle paths highly (both rating lanes higher than paths)
Tin Tin et al. (2010)	New Zealand	2469 cyclists, aged 16 years or over, who had enrolled in the 2006 Wattyl Lake Taupo Cycle Challenge	People aged 35+ (particularly over 50s) were more likely to report “more bicycle paths” would encourage them to cycle more	
Tiwari (2014)	India	Review including report of survey of current bicyclists and potential bicyclists in Pune, India		Preferences of females and males for bicycle routes showed similar trends except a few variables; differences were slight
Twaddle, Hall, and Bracic (2010)	Canada	Staff and students at University of Calgary, particularly potential or current cyclists	Females were more concerned than males about safety issues. Significant differences were found for “I do not know a safe route” and “I feel unsafe riding on roads” Males were more likely than females to indicate a desire for wide curb lanes [shared with general traffic]	There was no significant difference by gender in selection of any on-route improvements. Females were found to share similar preferences with males, high proportions of both wanting bicycle lanes, more pathways, and more direct routes.
Twaddle et al. (2010)	Canada	Staff and students at University of Calgary, particularly potential or current cyclists	Although all females were likely to indicate safety concerns prevent them from commuting by bicycle, the type of safety concern differs by age. Younger females are unsure about the route to take, with older females more concerned with feeling unsafe riding on the road	
Van Holle et al. (2014)	Belgium	59 middle-aged adults living in urban or semi-urban areas across Flanders and the Brussels Capital region	For observed characteristics included in the “choice task”: “evenness of the cycle path”, “presence of new elements”, “presence of historic elements” and “safety for crossing the street”, associations with proportion of invitingness for transportation cycling were only found in females, with results not significant in males	Moderating effects of gender on the association between the environmental characteristics and proportion of environmental invitingness for transportation cycling were absent in the final model for the choice task and the cognitive task
Vliet (2014)	The Netherlands	200 respondents from various parts of the Netherlands; mixed recruitment methods		Gender (and age) was not a significant factor in bicycle mode choice for short-distance commuting
Wardman, Tight, and Page (2007)	UK	1996 commuters in four English cities, having removed 60% judged never likely to contemplate cycling		No interaction effect between cycling-specific variables and gender or age
Westerdijk (1990)	UK/Sweden/Netherlands	284 cyclists and pedestrians aged 20+ in 3 countries (50 in Great Britain, 121 in Sweden and 113 in the Netherlands)		Distance, pleasantness and traffic safety were the most important attributes for cyclists, but few differences were found in the importance of attribute weights between male and female subjects, or older and younger groups
Winters and Teschke (2010)	Canada	1402 adult current and potential cyclists, that is, the “near market” for cycling in Vancouver, Canada	Females scored low preference routes even lower than males The two least preferred route types were major streets with no facilities, with or without parking (16% likely to choose). Only 79 respondents were “very likely” to choose to ride on major streets with parked cars. They represented a unique subpopulation: 22.6% regular cyclists (vs. 8.1% overall), mainly male (66.5%), aged 25–34, with a lower likelihood of having children (22.3% vs. 46.8%)	Virtually no differences in mean scores between males and females for the six most preferred route types (paved-off street paths for bikes only, paved off-street multiuse paths, unpaved off-street multi-use paths, cycle path next to major street separated by barrier, residential streets marked as bike routes with traffic calming, residential streets marked as bike routes)
Winters and Teschke (2010)	Canada	As above		Age was not in general a significant predictor of route choice preferences
Wooliscroft and Ganglmair-Wooliscroft (2014)	New Zealand	573 residents of New Zealand aged 18+		No statistical difference in the average part-worth rating of availability of cycle lanes by gender
Wooliscroft and Ganglmair-Wooliscroft (2014)	New Zealand	573 residents of New Zealand aged 18+	Age had some impact on the average part-worth of the availability of cycle lanes. Older respondents (over 50 years) assign them less utility than young respondents	Most results showed no significant differences by demographic group

### Other preferences

Studies highlighted some other similarities and differences by age and gender, but these proved too diverse to synthesise within the constraints of a systematic review. Research covered topics including preferences for cycling environments that minimise the impact of winter conditions (e.g. use of higher quality brine to maintain infrastructure), routes that are direct and ideally avoid hills, and routes that are well lit and overlooked.

## Discussion

We have found good evidence that women express stronger preferences for greater segregation from motor vehicles than men. This is within a context of similar overall types of preference, that is, typically very similar hierarchies of preference across genders. As stated by Misra, Watkins, and Le Dantec (2015): “Riders across all cyclist types prefer dedicated cycling facilities and are opposed to high speed traffic and high volume traffic, with little variation based on the classification of the cyclist”. In terms of age, again, there is an overall qualitative similarity between groups, but with some evidence suggesting that older people may have stronger preferences for separated infrastructure.

Gender differences were clearer among studies in low-cycling countries. In such settings, cycling is often perceived or experienced as risky, suitable only for the brave and confident (Horton & Jones, 2015). Men may be less concerned about risks than women, or more reticent about voicing their fears because these do not fit with dominant constructions of masculinity (Steinbach, Green, Datta, & Edwards, 2011). As such, the findings concerning gender differences in this review are arguably particularly relevant to places seeking to increase cycling from a low base.

Cycling speed may influence how views differ by age and gender. Cyclecraft, the UK’s national guide to cycling (Franklin, 2009), recommends a speed of 20 mph (32 kph) in challenging traffic situations. This is far faster than the average cycling speed, the gap being even greater for women and older people. Our analysis of National Travel Survey data^6^ indicates that in England, among those aged 18–29, the average speed was 11.3 mph men and 10.5 mph for women, while among those aged 60–69, it was 9.6 mph for men and 9 mph for women. Slower cyclists report more near misses per mile (Aldred and Crosweller, 2015). A stronger preference for separated infrastructure among older people could also stem from greater vulnerability to injury.

A few studies suggest that women may be more likely to be affected by barriers including the need to carry items, winter conditions, hills, and personal safety concerns (see also Damant-Sirois & El-Geneidy, 2015; Heinen, van Wee, & Maat, 2010). These issues merit further research, including how these factors might interact with infrastructural characteristics. Future stated preference work on gender could focus on the detail of infrastructure types (e.g. verge vs. kerb separated) and on how other factors, for example, cycling experience and cycling speed, affect preferences by gender. Another recommendation is for more research both on children’s own views, and on adult views about infrastructure for child cycling. Understanding how infrastructural change might impact child cycling is crucial not just for children but also for carers, disproportionately affecting trips made by women (Aldred et al., 2015).

Among studies covering age, definitions of older age varied considerably as did methods for evaluating its effects. While some studies used age in years as an independent variable within linear regression, or considered 3–6 categories, others used very different cut-offs for “older” cyclists. The use of harmonised age categories and/or treatment of age as a variable would have improved our ability to assess its impact. A recommendation that follows from this would be for stated preference studies to more routinely publish simple anonymised data sets (e.g. on the UK Data Archive or, given the non-sensitive nature of the data, on journal websites) suitable for individual-level meta-analysis. Comparability would also be enhanced by development of reporting guidelines.

The level of situational specificity varied substantially and this is worthy of further methodological investigation. Higher specificity potentially introduces more unobserved variation (e.g. related to path width or adjacent motor traffic), although this can be minimised or reduced (e.g. using manipulated photographs). However, higher specificity enables greater consistency in what people understand they are being asked to compare. Many less specific studies simply reference a “cycle lane”, which could be assumed to be either a painted on-road lane or track with varying levels of segregation (Steer Davies Gleave, 2010a, 2012). More realistic representation of infrastructure allows greater discrimination between options and may help us estimate more realistically the type of infrastructure that may be required to substantially grow cycling levels. While there is not one right way to do things, future research should aim for comparability with published methods wherever possible. This is not to deny the need for innovation. Future research into infrastructure preferences may want to consider combining qualitative and quantitative approaches, and make greater use of video methods (see e.g. Ghekiere et al., 2014, 2015).

Finally, policy should focus on the infrastructural needs and preferences of under-represented groups, including older people, women, children and those cycling with children or making decisions about child cycling. Younger people, men, and those travelling without children also generally prefer separation from motor traffic, so building for under-represented groups should, if done well, suit others. Inclusive infrastructure is particularly important given evidence that some other barriers to cycling may be stronger for under-represented groups (van Bekkum, 2011; Bergström & Magnusson, 2003; Daley, Rissel, & Lloyd, 2007; Damant-Sirois & El-Geneidy, 2015; Finch et al., 1985; Steinbach et al., 2011). For example, women may have stronger concerns than men about safety from crime, while older people may struggle to cycle longer distances. Focusing on the needs and preferences of under-represented groups should be sensitive to these issues and, for example, take account of concerns about crime and route directness when planning the location of high-quality infrastructure.

## Supplementary Material

TTRV_1200156_Appendix_Material.docx

## Notes

1. Definitions of “non-cyclists” varied between studies; here, we mean someone who did not cycle in the last week (or longer). It was not always possible to determine whether there were over 20% non-cyclists or not.

2. Note: in the case of the 17 publications where full text could not be found, both reviewers read the abstracts and considered almost all unlikely to be relevant: most were conference papers or policy reports, and often seemed relatively tangentially connected to the research question (we had erred on the side of inclusivity in Stage 3 where we could not initially find publications).

3. In two cases, graphs were given illustrating this, but not the precise figures.

4. One study did not report sample size.

5. Not all studies could be included due to difficulty in determining numbers of cyclists and non-cyclists.

6. 
https://discover.ukdataservice.ac.uk/series/?sn=2000037.
