# Supplementary material for: Cycling provision separated from motor traffic: a systematic review exploring whether stated preferences vary by gender and age
Source: Transp Rev. 2016 Jul 14;37(1):29–55. doi: 10.1080/01441647.2016.1200156 (PMC5259802; doi:10.1080/01441647.2016.1200156)
Supplement: TTRV_1200156_Appendix_Material.docx [file ttrv_a_1200156_sm7021.docx]

## Appendix: Further Details of Protocol and Methods

### Search terms

The research question was structured using the PICO - (Population, Intervention, Comparison, and Outcome) framework - and used to determine key search terms. These are summarised in Table 1, while a more comprehensive outline of searches and records retrieved is available at the end of this appendix.

The search terms and search strategy, developed with advice from researchers with expertise in conducting systematic reviews about cycling, sought to reflect terms commonly used in relevant academic and policy papers, which varies by country context (for example ‘bicycle boulevard’ is widely used in US literature, but not in the UK). As policy literature often highlights the presence of children as significant for infrastructure preferences (see e.g. DfT 2008), the review sought to cover preferences related to cycling with or by children.

Table 1: Summary of key search terms used

**P terms**: bike$ OR bicycle$ OR bicycling OR bicyclist$ OR cycle OR cyclist$ OR cycling OR “active travel” OR “active transport” OR “non-motorised modes” OR “non-motorised transport” OR “non-motorized modes” OR “non-motorized transport”

AND

**I terms:** infrastructure OR track$ OR lane$ OR “off-road” OR “off-street” OR “on-road” OR “on-street” OR junction$ OR box OR ASL OR “traffic calming” OR “traffic reduction” OR “traffic removal” OR boulevard$ OR filter* OR “road closure” OR greenway$ OR residential OR segregat* OR protected OR painted OR path$ OR facility OR facilities

AND

**C terms: sex OR gender$ OR age* OR children OR men OR women OR male$ OR female$ OR older OR younger OR elderly**

AND

**O terms:** prefer* OR choice$ OR choosing OR decision$ OR attitud* OR view* OR willing*

### Databases and websites searched

A number of databases and websites were searched to retrieve relevant online material and results downloaded to reference-management software Mendeley. To guide the searches, include/exclude criteria regarding relevance, setting, quality, date, format and language were determined:

Table 2: Inclusion and Exclusion Criteria for relevance, setting, quality, date, format, language

|  | **Include in Review** | **Exclude** |
| --- | --- | --- |
| **Relevance** | Evidence relating to stated preferences (what people say about their preferences, and/or how a particular intervention might change their behaviour)  Evidence that specifically relates to route infrastructure preferences (defined broadly as including e.g. lighting and maintenance as well as type of road infrastructure) | Evidence related to observed behaviour  Evidence on views about other policies, e.g. financial incentives, provision of hire bicycles  Views about barriers to cycling (as opposed to views about infrastructure types). |
| **Setting** | Any country |  |
| **Quality** | Peer reviewed academic literature; government-commissioned literature; grey literature, i.e. working papers, NGO, think-tank and consultant reports | Unreferenced, non-traceable web reports |
| **Date of research** | Published in the last 25 years | Published more than 25 years ago |
| **Format** | Available electronically | Only available in print form / electronic version no longer available and cannot be sourced from author/ stakeholders |
| **Language** | English only | Non-English |

#### Databases

The following databases were searched. The initial requirement had been for one of the search terms to be ‘title-only’, in order to reduce the numbers of irrelevant results, generated due to the multiple meanings of the words ‘cycle’ and ‘cycling’. Initial investigation suggested that the TRID (specialist) database was by some way the most relevant, and the generation of irrelevant results less of a problem. We therefore adapted our search strategy for TRID, removing the requirement for one of the search terms to be ‘title-only’, so conducting a somewhat broader search.

- EBSCO (Business Source Complete, EconLit, Greenfile, Medline, REPEC) (*170 results*)
- Web of Science (*121 results*)
- ProQuest Dissertations & Theses: UK & Ireland (*21 results*)
- PubMed (*41 results*)
- TRID (including ITS) (*573 results*)
- ARRB Knowledge Base (*10 results*)

This yielded 936 peer-reviewed journal papers. Three more databases were originally proposed for searching (International Transport Forum, REPEC and Scopus). Two of these are incorporated within EBSCO and TRID and so did not require separate searching. The reviewers did not have access to Scopus as the University of Westminster instead subscribes to Web of Science, its main competitor.

#### Websites

The following websites were searched using Google’s advanced search facility, to ensure a consistent search approach.

- Danish Transport Research Institute- [www.transport.dtu.dk/english](http://www.transport.dtu.dk/english)
- National Highway Traffic Safety Administration (USA) - [www.nhtsa.dot.gov](http://www.nhtsa.dot.gov)
- New York City Department of Transportation-[www.nyc.gov/html/dot/html/home/home.shtml](http://www.nyc.gov/html/dot/html/home/home.shtml)
- OpenSIGLE-<http://opensigle.inist.fr>
- Pedestrian and Bicycle Information Center- [www.bicyclinginfo.org/](http://www.bicyclinginfo.org/)
- Portland Department of Transportation-[www.portlandoregon.gov/transportation/](http://www.portlandoregon.gov/transportation/)
- Swedish Transport Administration- [www.trafikverket.se](http://www.trafikverket.se)
- Transport Canada- [www.tc.gov](http://www.tc.gov)
- Transport for London-[www.tfl.gov.uk](http://www.tfl.gov.uk)
- Transport Research Laboratory (UK) - [www.trl.co.uk](http://www.trl.co.uk)
- UK Department for Transport-[www.gov.uk/government/organisations/department-for-transport](http://www.gov.uk/government/organisations/department-for-transport)

Due to the large number of results retrieved for some websites (in some cases, thousands), we decided to include only the first twenty PDF documents produced by the search for each site; i.e. 220 documents. This is in line with practice elsewhere, for example Oh et al 2005 who included the top 400 Google documents found. Google's algorithm works by searching out Web pages including the keywords used, assigning a rank to each page based on factors including how many times the keywords appear. On examining the 220 documents, some were clearly irrelevant (e.g. about motorcycling) and only those with potential relevance were imported into Mendeley.

Website searches were complemented by a Google Scholar search. This searched abstracts of any publications added but only in the past year, due to the limits of Google Scholar functionality. This helped in accessing new publications not yet added to other indexing databases.

The web searches yielded a total of 176 additional publications, after removing duplicates. Finally, the lead author was aware of five additional relevant publications (including one that remains unpublished, and two unpublished then) which were included.

### Information recorded during appraisal

| Item | Details |
| --- | --- |
| Country of study | Then also categorised depending on income levels and cycling levels |
| Population characteristics | Including a qualitative description |
| Sample size | Including numbers of cyclists |
| Sampling approach | Whether convenience sampling, random sampling or purposive sampling. |
| Situations presented | A short description of the situations presented to participants |
| Situational specificity | How specific were the situations presented to respondents, on a 1-3 scale |
| Elicitation method | How participants were asked about situations, e.g. text only or also images |
| Groups covered | Whether differences/similarities were discussed in relation to age, gender, and/or children. |
| Summary of findings | A brief description of the findings, focusing on reported differences or similarities in cycle route infrastructure preferences by age and gender. We considered differences to exist if the differences were reported by study authors as statistically significant. |

### Further details of screening

**Screening Round 1:** The searches of the databases and online resources together captured **1117** separate publications (after removing duplicates) and the citations for each were uploaded into Mendeley. Most of these included full abstracts. The first round of screening was carried out, based on a quick review of the titles only and removed any material which a) did not have anything to do with riding bicycles at all (e.g. chemical cycles) b) was only concerned with sport cycling/ indoor exercise cycling/round the world cycle trips or c) focused on the mechanics of bicycle design/ the structure of the bicycle industry.

BE carried out the initial screening for Round 1. RA checked all decisions and adjusted the codes inclusively (i.e. to minimise unnecessary exclusions). **153** publications were excluded.

**Screening Round 2:** The second round of screening involved a review of the titles and abstracts for each of the **964** remaining publications. Publications not excluded from the study at this stage included those where the reviewer thought that there may be some attempt to measure expressed preferences for different types of cycle infrastructure or environment, regardless of whether age /gender contrasts were apparent at this stage. Examples of material excluded from the study at this stage included a) where a publication related only to bicycle related injuries (rather than, for example, perceptions of safety) b) where a publication was clearly reporting only actual cycling behaviour (e.g. where people go) and not expressed preferences (what people say) and c) where a publication related only to attitudes to/use of safety clothing e.g. bicycle helmets.

BE carried out the initial screening for Round 2.RA checked all decisions and adjusted the codes inclusively (i.e. to minimise unnecessary exclusions), **662** publications were excluded.

**Screening Round 3:** We then reviewed the titles, abstracts, and (in just under 50% of cases) full text of the remaining **302** publications and tagging them either for inclusion or exclusion for one of the following reasons:

- No infrastructure preferences measured: not a publication including information about preferences for different route characteristics. This excluded studies where, for example, the research only looked at preferences for hire bicycle availability. It also excluded publications where the research reported focused on correlations between cycling levels and beliefs about the cycling environment (e.g. that there were enough cycle lanes, or whether people feel cycling is safe). (110 publications)
- No age/gender differences presented: where the publication seemed to cover relevant ground but there was no information about any differences or lack of differences by age or by gender. (65 publications)
- Other: this included instances where there were issues with the publication (e.g. full text not in English, source was a presentation not a paper or report) (15 publications)

BE carried out the initial screening for Round 3, following which RA independently reviewed all publications. In 146 cases, this also involved rapid reading of full text sources, where there was disagreement or uncertainty about whether to include the publication, provided it was available online. This additional full text reading was done primarily to minimise the extent to which some publications might be missed due to the abstract not reporting having examined differences by age and gender. Screening Round 3 led to the removal of **190** publications.

**Screening round 4** involved 112 publications. For articles where no full text had been available, authors were contacted. Full text was obtained for 95/112 publications and read by both RA and BE. The 17 excluded papers were very unlikely to be relevant, but had been left in up till that stage so as not to exclude anything potentially relevant without having viewed the full text. Of the 95 publications, 37 were then excluded as not answering the research question, leaving 58 publications included in the appraisal. These represented **54 studies**.

### **Full details of search terms used**

**P terms**: bike$ OR bicycle$ OR bicycling OR bicyclist$ OR cycle OR cyclist$ OR cycling OR “active travel” OR “active transport” OR “non-motorised modes” OR “non-motorised transport” OR “non-motorized modes” OR “non-motorized transport” OR ride* OR riding

(NOT “life-cycle” OR “life-cycles” OR “menstrual-cycle” OR “menstrual-cycles” OR “menstrual-cycling” OR “cycle-phase” OR “regulated-cycle” OR “ulster-cycle” OR “amadis-cycle” OR “earthquake-cycle” OR “world-cycles” OR “christi-cycles” OR “cardiac-cycle” OR “reproduction-cycle” OR “reproductive-cycle” OR “aging-cycle” OR “sexual-cycle” OR “water-cycle” OR “seasonal-cycle” OR “cell-cycle” OR “night-cycle” OR “day-cycle” OR “isotopes-cycle” OR “hormonal-cycle” OR “vicious-cycle” OR “sea-level cycle” OR “feeding-cycle” OR “estrous-cycle” OR “estrus-cycle” OR “second-cycle” OR “hidden-cycle” OR “the-cycle” OR “cycle-of” OR “business-cycle” OR “business-cycles” OR “astrand-cycle” OR “Krebs-cycle” OR “poverty-disease-cycle” OR “career-cycle” OR “extended-cycle” OR “fuel-cycle” OR “treatment-cycle” OR “gait-cycle” OR “modelling cycle” OR “production-cycle”)

AND

**I terms:** infrastructure OR track$ OR lane$ OR “off-road” OR “off-street” OR “on-road” OR “on-street” OR junction$ OR box OR ASL OR “traffic calming” OR “traffic reduction” OR “traffic removal” OR boulevard$ OR filter* OR “road closure” OR greenway$ OR residential OR segregat* OR protected OR painted OR path$ OR facility OR facilities

AND

**C terms: sex OR gender$ OR age* OR children OR men OR women OR male$ OR female$ OR older OR younger OR elderly**

AND

**O terms:** prefer* OR choice$ OR choosing OR decision$ OR attitud* OR view* OR willing*

**Summary of literature searches**

| **Resources (in order searched)** | **Description** | **Date searched** | **Search terms** | **No. of records retrieved** |
| --- | --- | --- | --- | --- |
| **EBSCO (Business Source Complete, EconLit, Greenfile, Medline)** | Business Source Complete- over 10,000 full text journals covering all areas of business. EconLit- Index of journal articles, books and working papers in all areas of economics. Greenfile- Index to journal articles on all aspects of human impact to the environment. Medline- Index of journal articles covering a range of medical topics relating to research, clinical practice, policy, and health care services. | 12 March | TI ( bike OR bicycle OR bicycling OR bicyclist OR cycle OR cyclist OR cycling OR “active travel” OR “active transport” OR “non-motorised modes” OR “non-motorised transport” OR “non-motorized modes” OR “non-motorized transport”) NOT TI ( see above ) AND TI ( sex OR gender OR age* OR children OR men OR women OR male OR female OR older OR younger OR elderly ) AND TI ( prefer* OR choice OR choosing OR decision OR attitud* OR view* OR willing* )  TI ( bike OR bicycle OR bicycling OR bicyclist OR cycle OR cyclist OR cycling OR “active travel” OR “active transport” OR “non-motorised modes” OR “non-motorised transport” OR “non-motorized modes” OR “non-motorized transport”) NOT TI (see above ) AND TI ( infrastructure OR track OR lane OR “off-road” OR “off-street” OR “on-road” OR “on-street” OR junction OR box OR ASL OR “traffic calming” OR “traffic reduction” OR “traffic removal” OR boulevard OR filter* OR “road closure” OR greenway OR residential OR segregat* OR protected OR painted OR path OR facility OR facilities ) AND TI ( prefer* OR choice OR choosing OR decision OR attitud* OR view* OR willing* )  TI ( bike OR bicycle OR bicycling OR bicyclist OR cycle OR cyclist OR cycling OR “active travel” OR “active transport” OR “non-motorised modes” OR “non-motorised transport” OR “non-motorized modes” OR “non-motorized transport”) NOT TI (see above ) AND TI ( infrastructure OR track OR lane OR “off-road” OR “off-street” OR “on-road” OR “on-street” OR junction OR box OR ASL OR “traffic calming” OR “traffic reduction” OR “traffic removal” OR boulevard OR filter* OR “road closure” OR greenway OR residential OR segregat* OR protected OR painted OR path OR facility OR facilities ) AND TI ( sex OR gender OR age* OR children OR men OR women OR male OR female OR older OR younger OR elderly )  TI (bike OR bicycle OR bicycling OR bicyclist OR cycle OR cyclist OR cycling OR “active travel” OR “active transport” OR “non-motorised modes” OR “non-motorised transport” OR “non-motorized modes” OR “non-motorized transport”) NOT TI (see above) AND AB (sex OR gender OR age* OR children OR men OR women OR male OR female OR older OR younger OR elderly) AND AB (prefer* OR choice OR choosing OR decision OR attitud* OR view* OR willing*)  TI (bike OR bicycle OR bicycling OR bicyclist OR cycle OR cyclist OR cycling OR “active travel” OR “active transport” OR “non-motorised modes” OR “non-motorised transport” OR “non-motorized modes” OR “non-motorized transport”) NOT TI (see above) AND AB (infrastructure OR track OR lane OR “off-road” OR “off-street” OR “on-road” OR “on-street” OR junction OR box OR ASL OR “traffic calming” OR “traffic reduction” OR “traffic removal” OR boulevard OR filter* OR “road closure” OR greenway OR residential OR segregat* OR protected OR painted OR path OR facility OR facilities) AND AB (sex OR gender OR age* OR children OR men OR women OR male OR female OR older OR younger OR elderly)  TI (bike OR bicycle OR bicycling OR bicyclist OR cycle OR cyclist OR cycling OR “active travel” OR “active transport” OR “non-motorised modes” OR “non-motorised transport” OR “non-motorized modes” OR “non-motorized transport”) NOT TI (see above) AND AB (infrastructure OR track OR lane OR “off-road” OR “off-street” OR “on-road” OR “on-street” OR junction OR box OR ASL OR “traffic calming” OR “traffic reduction” OR “traffic removal” OR boulevard OR filter* OR “road closure” OR greenway OR residential OR segregat* OR protected OR painted OR path OR facility OR facilities) AND AB (prefer* OR choice OR choosing OR decision OR attitud* OR view* OR willing*)  **Total records (without duplicates)** | 2  5  2  47  78  103  **170** |
| **Web of Science** | Indexes of journal articles and more, including Web of Science (Science Citation Index, Social Sciences Citation Index, Arts & Humanities Citation Index), ISI Proceedings, MEDLINE and Journal Citation Reports. |  | (bike$ OR bicycle$ OR bicycling OR bicyclist*OR cycle OR cyclist* OR cycling OR active travel OR active transport OR non-motorised modes OR non-motorised transport OR non-motorized modes OR non-motorized transport) NOT TOPIC: (see above) AND TOPIC: (infrastructure OR track$ OR lane$ OR “off-road” OR “off-street” OR “on-road” OR “on-street” OR junction$ OR box OR ASL OR “traffic calming” OR “traffic reduction” OR “traffic removal” OR boulevard$ OR filter* OR “road closure” OR greenway$ OR residential OR segregat* OR protected OR painted OR path$ OR facility OR facilities) AND TOPIC: (sex OR gender$ OR age OR children OR men OR women OR male OR female OR older OR younger OR elderly) AND TOPIC: (prefer* OR choice* OR choosing OR decision OR attitud* OR view* OR willing*) | 121 |
| **ProQuest Dissertations & Theses - UK and Ireland** | A comprehensive listing of theses with abstracts accepted for higher degrees by universities in the United Kingdom and Ireland since 1716. Links to full-text where available online. |  | ti(bike OR bicycle OR bicycling OR bicyclist OR cycle OR cyclist OR cycling OR "active travel" OR "active transport" OR "non-motorised modes" OR "non-motorised transport" OR "non-motorized modes" OR "non-motorized transport") AND ab(sex OR gender OR age OR children OR men OR women OR male OR female OR older OR younger OR elderly) AND ab(prefer* OR choice OR choosing OR decision OR attitude* OR view* OR willing*) NOT ti,ab(see above)  ti(bike OR bicycle OR bicycling OR bicyclist OR cycle OR cyclist OR cycling OR "active travel" OR "active transport" OR "non-motorised modes" OR "non-motorised transport" OR "non-motorized modes" OR "non-motorized transport") AND ab(infrastructure OR track OR lane OR "off-road" OR "off-street" OR "on-road" OR "on-street" OR junction OR box OR ASL OR "traffic calming" OR "traffic reduction" OR "traffic removal" OR boulevard OR filter* OR "road closure" OR greenway OR residential OR segregat* OR protected OR painted OR path OR facility OR facilities) AND ab(sex OR gender OR age OR children OR men OR women OR male OR female OR older OR younger OR elderly) NOT ti,ab(see above)  ti(bike OR bicycle OR bicycling OR bicyclist OR cycle OR cyclist OR cycling OR "active travel" OR "active transport" OR "non-motorised modes" OR "non-motorised transport" OR "non-motorized modes" OR "non-motorized transport") AND ab(infrastructure OR track OR lane OR "off-road" OR "off-street" OR "on-road" OR "on-street" OR junction OR box OR ASL OR "traffic calming" OR "traffic reduction" OR "traffic removal" OR boulevard OR filter* OR "road closure" OR greenway OR residential OR segregat* OR protected OR painted OR path OR facility OR facilities) AND ab(prefer* OR choice OR choosing OR decision OR attitude* OR view* OR willing*) NOT ti,ab(see above)  ti(bike OR bicycle OR bicycling OR bicyclist OR cycle OR cyclist OR cycling OR "active travel" OR "active transport" OR "non-motorised modes" OR "non-motorised transport" OR "non-motorized modes" OR "non-motorized transport") AND ab,ft(sex OR gender OR age OR children OR men OR women OR male OR female OR older OR younger OR elderly) AND ab,ft(prefer* OR choice OR choosing OR decision OR attitude* OR view* OR willing*) NOT ti,ab,ft(see above)  **Total records (without duplicates)** | 8  4  11  10  21 |
| PubMed | 300,000+ references are added annually from 3,000+ international journals. Equivalent to INDEX MEDICUS. References with abstracts to biomedical literature including research and clinical practice, administration, policy issues and health care services. |  | (((((bike[Title] OR bikes[Title] OR bicycle[Title] OR bicycles[Title] OR bicycling[Title] OR bicyclist*[Title] OR cycle[Title] OR cyclist*[Title] OR cycling[Title] OR “active travel”[Title] OR “active transport”[Title] OR “non-motorised modes”[Title] OR “non-motorised transport”[Title] OR “non-motorized modes”[Title] OR “non-motorized transport”[Title])) NOT (see above)) AND (infrastructure[Title/Abstract] OR track[Title/Abstract] OR tracks[Title/Abstract] OR lane[Title/Abstract] OR lanes[Title/Abstract] OR “off-road”[Title/Abstract] OR “off-street”[Title/Abstract] OR “on-road”[Title/Abstract] OR “on-street”[Title/Abstract] OR junction[Title/Abstract] OR junctions[Title/Abstract] OR box[Title/Abstract] OR "ASL"[Title/Abstract] OR “traffic calming”[Title/Abstract] OR “traffic reduction”[Title/Abstract] OR “traffic removal”[Title/Abstract] OR boulevard[Title/Abstract] OR boulevards[Title/Abstract] OR filter*[Title/Abstract] OR “road closure”[Title/Abstract] OR greenway[Title/Abstract] OR greenways[Title/Abstract] OR residential[Title/Abstract] OR segregat*[Title/Abstract] OR protected[Title/Abstract] OR painted[Title/Abstract] OR path[Title/Abstract] OR paths[Title/Abstract] OR facility[Title/Abstract] OR facilities[Title/Abstract])) AND (sex[Title/Abstract] OR gender[Title/Abstract] OR genders[Title/Abstract] OR age[Title/Abstract] OR aged[Title/Abstract] OR ages[Title/Abstract] OR child*[Title/Abstract] OR men[Title/Abstract] OR women[Title/Abstract] OR male[Title/Abstract] OR males[Title/Abstract] OR female[Title/Abstract] OR females[Title/Abstract] OR older[Title/Abstract] OR younger[Title/Abstract] OR elderly[Title/Abstract])) AND (prefer*[Title/Abstract] OR choice[Title/Abstract] OR choices[Title/Abstract] OR choosing[Title/Abstract] OR decision[Title/Abstract] OR decisions[Title/Abstract] OR attitud*[Title/Abstract] OR view*[Title/Abstract] OR willing*[Title/Abstract]) Filters: Publication date from 1990/01/01 to 2015/12/31  **Total records (without duplicates)** | 41 |
| TRID | TRID is a newly integrated database that combines the records from TRB's Transportation Research Information Services (TRIS) Database and the OECD's Joint Transport Research Centre’s International Transport Research Documentation (ITRD) Database. TRID provides access to over 900,000 records of transportation research worldwide. |  | You searched with subject: **Pedestrians and Bicyclists** and with index term: **Bicycle facilities, Bikeways, Bicycle commuting, Bicycle lanes, Bicycle travel, Sidewalks, Traffic calming, Infrastructure, Filters, Greenways, Segregation (Aggregates), Facilities, Environment** and with result type: **Articles and papers** with keywords containing **prefer* OR choice* OR choosing OR decision* OR attitud* OR view* OR willing*** and with title containing **bike* OR bicycle* OR bicycling OR bicyclist* OR bicyclists' OR cycle OR cyclists OR cycling OR “active travel” OR “active transport” OR “non-motorised modes” OR “non-motorised transport” OR “non-motorized modes” OR “non-motorized transport” OR ride* OR riding** between dates **1990 – 2015**  You searched with subject: **Pedestrians and Bicyclists** and with index term: **Bicycle facilities, Bikeways, Bicycle commuting, Bicycle lanes, Bicycle travel, Sidewalks, Traffic calming, Infrastructure, Filters, Greenways, Segregation (Aggregates), Facilities, Environment** and with result type: **Articles and papers** with keywords containing **sex OR gender* OR age* OR child* OR men OR women OR male* OR female* OR older OR younger OR elderly** and with title containing **bike* OR bicycle* OR bicycling OR bicyclist* OR bicyclists' OR cycle OR cyclists OR cycling OR “active travel” OR “active transport” OR “non-motorised modes” OR “non-motorised transport” OR “non-motorized modes” OR “non-motorized transport” OR ride* OR riding** between dates **1990 - 2015**  **Total records (without duplicates)** | 573 |
| ARRB Knowledge Base |  |  | **P and C terms and P and O terms**  **Total records (without duplicates)** | 10 |

**Summary of grey literature searches**

| **Resources (in order searched)** | **Description** | **Date searched** | **Search terms** | **No. of records retrieved (first 2 pages or 20 pdfs imported to Mendeley)** |
| --- | --- | --- | --- | --- |
| Danish Transport Research Institute | www.transport.dtu.dk | 20 March | (bike OR bicycle OR bicycling OR bicyclist OR cycling OR cyclist OR "active transport" OR "active travel" OR "non-motorised modes") (sex OR gender OR age OR child OR male OR female OR older OR younger OR elderly) (prefer OR choice OR decision OR attitude OR view OR willing) site:www.transport.dtu.dk filetype:ashx | 27 |
| Department for Transport (UK) | www.gov.uk | 23 March | (bike OR bicycle OR bicycling OR bicyclist OR cycling OR cyclist OR "active transport" OR "active travel" OR "non-motorised modes") (sex OR gender OR age OR child OR male OR female OR older OR younger OR elderly) (prefer OR choice OR decision OR attitude OR view OR willing) site:www.gov.uk filetype:pdf | 3,340 |
| New York City Department of Transportation | www.nyc.gov | 23 March | (bike OR bicycle OR bicycling OR bicyclist OR cycling OR cyclist OR "active transport" OR "active travel" OR "non-motorised modes") (sex OR gender OR age OR child OR male OR female OR older OR younger OR elderly) (prefer OR choice OR decision OR attitude OR view OR willing) site:www.nyc.gov filetype:pdf | 2,820 |
| National Highway Traffic Safety Administration | www.nhtsa.dot.gov | 23 March | (bike OR bicycle OR bicycling OR bicyclist OR cycling OR cyclist OR "active transport" OR "active travel" OR "non-motorised modes") (sex OR gender OR age OR child OR male OR female OR older OR younger OR elderly) (prefer OR choice OR decision OR attitude OR view OR willing) site:www.nhtsa.dot.gov filetype:pdf | 47 |
| Pedestrian and Bicycle Information Centre | www.bicyclinginfo.org | 20 March | (bike OR bicycle OR bicycling OR cycling OR cyclist OR "active transport" OR "active travel" OR "non-motorised modes") (sex OR gender OR age OR child OR male OR female OR older OR younger OR elderly) (prefer OR choice OR decision OR attitude OR view OR willing) site:www.bicyclinginfo.org filetype:pdf | 2 |
| Portland Department of Transportation | www.portlandoregon.gov/transportation | 23 March | (bike OR bicycle OR bicycling OR bicyclist OR cycling OR cyclist OR "active transport" OR "active travel" OR "non-motorised modes") (sex OR gender OR age OR child OR male OR female OR older OR younger OR elderly) (prefer OR choice OR decision OR attitude OR view OR willing) site:www.portlandoregon.gov/transportation | 572 |
| Swedish Transport Administration | www.trafikverket.se | 20 March | (bike OR bicycle OR bicycling OR bicyclist OR cycling OR cyclist OR "active transport" OR "active travel" OR "non-motorised modes") (sex OR gender OR age OR child OR male OR female OR older OR younger OR elderly) (prefer OR choice OR decision OR attitude OR view OR willing) site:http://www.trafikverket.se filetype:pdf | 24 |
| TC- Transport Canada | www.tc.gc.ca | 20 March | (bike OR bicycle OR bicycling OR bicyclist OR cycling OR cyclist OR "active transport" OR "active travel" OR "non-motorised modes") (sex OR gender OR age OR child OR male OR female OR older OR younger OR elderly) (prefer OR choice OR decision OR attitude OR view OR willing) site:www.tc.gc.ca filetype:pdf | 54 |
| Transport for London | www.tfl.gov.uk | 20 March | (bike OR bicycle OR bicycling OR bicyclist OR cycling OR cyclist OR "active transport" OR "active travel" OR "non-motorised modes") (sex OR gender OR age OR child OR male OR female OR older OR younger OR elderly) (prefer OR choice OR decision OR attitude OR view OR willing) site:www.tfl.gov.uk filetype:pdf | 806 |
| TRL- Transport Research Laboratory (UK) | www.trl.co.uk | 23 March | (bike OR bicycle OR bicycling OR bicyclist OR cycling OR cyclist OR "active transport" OR "active travel" OR "non-motorised modes") (sex OR gender OR age OR child OR male OR female OR older OR younger OR elderly) (prefer OR choice OR decision OR attitude OR view OR willing) site:www.trl.co.uk filetype:pdf | 807 |
| Google scholar (last year) |  | 20 March | (bike OR bicycle OR bicycles OR cyclist OR cycling OR "active transport" OR "active travel" OR "non-motorised modes") (sex OR gender OR age OR child OR male OR female OR older OR younger) (preference OR choice OR decision OR attitude OR view OR willing) Articles added in the last year, sorted by date (searching abstracts) | 117 |
| OpenSigle | www.opengrey.eu | 23 March | (bike OR bicycle OR bicycling OR bicyclist OR cycling OR cyclist OR "active transport" OR "active travel" OR "non-motorised modes") AND (sex OR gender OR age OR child OR prefer OR choice OR decision OR attitude OR view OR willing) discipline:(05V - Urban planning, rural planning, transport planning, countryside conservation) | 13 |
